# Process evaluation of the Walk Well study: a cluster-randomised controlled trial of a community based walking programme for adults with intellectual disabilities

**DOI:** 10.1186/s12889-016-3179-6

**Published:** 2016-07-07

**Authors:** Lynsay Matthews, Fiona Mitchell, Kirsten Stalker, Alex McConnachie, Heather Murray, Chris Melling, Nanette Mutrie, Craig Melville

**Affiliations:** Institute of Health and Wellbeing, MRC/CSO Social and Public Health Sciences Unit, University of Glasgow, Glasgow, Scotland; Department of Physical Activity for Health, University of Strathclyde, Glasgow, Scotland; Glasgow School of Social Work, University of Strathclyde, Glasgow, Scotland; Institute of Health and Wellbeing, Robertson Centre for Biostatistics, University of Glasgow, Glasgow, Scotland; Social Work Services, Glasgow City Council, Glasgow, Scotland; Institute for Sport, Physical Education and Health Sciences, Moray House School of Education, University of Edinburgh, Edinburgh, Scotland; Institute of Health and Wellbeing, Academic Unit for Mental Health & Wellbeing, University of Glasgow, Glasgow, Scotland

**Keywords:** Process evaluation, Intellectual disabilities, Physical activity, Walking, Behaviour change, Pedometers, Randomised-controlled trial

## Abstract

**Background:**

Walking interventions can be effective in encouraging sedentary populations to become more active; however, limited research has explored the effectiveness of walking interventions for adults with intellectual disabilities. This process evaluation explored the delivery of a community based walking intervention for adults with intellectual disabilities.

**Methods:**

Walk Well was a single-blind cluster randomised controlled trial of a 12-week physical activity consultation-led walking intervention. 102 participants were randomised to the Walk Well intervention or a waiting list control group. Participants in the intervention group received three physical activity consultations with a walking advisor at baseline, 6 & 12-weeks. They were encouraged to use a pedometer to set goals and monitor their daily step count. Primary outcome was change in daily step count at 12-weeks. Process evaluation measures included qualitative interviews with key stakeholders (*n* = 6) and quantifiable data collected as part of the intervention. Additional process data were extracted from a sub-set of qualitative interviews with participants and carers (*n* = 20). Data were analysed for process information related to context, recruitment and retention, reach, implementation, and fidelity.

**Results:**

Walk Well was not effective in significantly increasing levels of physical activity. The process evaluation did, however, highlight several important areas for consideration in future studies, including: a successful recruitment and retention strategy reaching a representative sample of adults with intellectual disabilities in the community; feasible and (for most) enjoyable methods of engaging adults with intellectual disabilities in activities to support behaviour change; potential need for greater intervention duration and frequency of contact; advantages and disadvantages of using pedometers as a behaviour change tool; the need for strategies which engage carers in supporting participants; and the complex issue of ‘freedom of choice’ in relation to lifestyle behaviours and study participation.

**Conclusions:**

Walking interventions for adults with intellectual disabilities can be feasibly delivered in the community in relation to reach, recruitment, retention and intervention fidelity. More intensive intervention methods need to be explored as well as strategies to engage and motivate carers in their support of participants.

**Trial registration:**

Current Controlled Trials ISRCTN50494254 (3^rd^ April 2012).

## Background

Adults with intellectual disabilities have significant health inequalities when compared with the general population [1]. They have higher rates of mortality [2, 3], obesity [4, 5], and experience greater physical and mental health needs [6, 7]. As well as the implications for individual health, these inequalities translate into a significant challenge for both health and social services [8].

Increased barriers to physical activity lead the majority of adults with intellectual disabilities to be physically inactive and engage in sedentary behaviour [9, 10]. They are significantly less active than adults without intellectual disabilities [11, 12], with one study suggesting individuals with intellectual disabilities walk on average 15 minutes per week [11].

Walking is widely acknowledged as a feasible activity to engage inactive and sedentary individuals in physical activity [13]. It requires minimal training, resources or expense [14] and can be an appropriate activity for adults with a range of intellectual disabilities. It is encouraged as a method of achieving the current physical activity guidelines of 150 minutes of moderate activity per week [15]. Activity can be accumulated in minimum bouts of 10-minutes or more, making short episodes of walking a feasible way of integrating physical activity into daily life [15].

One recommended step target for healthy adults is 10,000 steps per day [16]. Research suggests that adults with a disability or chronic illness should aim for a reduced daily step count of 6500-8500 steps per day, with 3000 of these steps performed at moderate to vigorous intensity [17]. Despite these recommendations there is limited research exploring the intensity or cadence of walking for adults with intellectual disabilities. Previous studies suggest that adults with intellectual disabilities walk between 6481 and 11,101 steps per day [11, 18, 19]. This wide range in daily step count may reflect a recruitment bias, where individuals who were already active were more likely to participate in the physical activity interventions [20].

Walking is the most common form of physical activity in adults with intellectual disabilities [11]. However, despite its feasibility and potential for health benefits only one study has published findings from a walking intervention [21]. Moss (2009) delivered a 12-week walking intervention to one hundred adults with intellectual disabilities. Participants walked three times per week around the grounds of the residential institution where they lived, with an increasing duration of 20-30minutes over the course of the 12-week intervention. Participants increased their level of physical activity and reduced their percentage of body fat, demonstrating the potential of walking as a method of eliciting health benefits in adults with intellectual disabilities. The relevance of this one study was limited by the institutional setting, its lack of follow-up and comparison with a control group. There is therefore a research gap to be addressed in the effectiveness of walking interventions for adults with intellectual disabilities.

This paper presents process evaluation findings from the *Walk Well* intervention, a randomised controlled trial (RCT) aimed at promoting physical activity via a 12-week evidence based walking progamme. The protocol and main findings of Walk Well have been published elsewhere [22, 23]. This complementary paper explores the processes and potential mechanisms of impact of the intervention. Process evaluations are one such method, as recommended by the Medical Research Council [24] and World Health Organisation [25]. They enable researchers to identify elements of intervention delivery which were effective or ineffective, and under what circumstances [26]. Publication of process data also informs the development of similar interventions by other researchers or service providers.

### Aim

The aim of this process evaluation was to explore the feasibility of a 12-week walking intervention for adults with intellectual disabilities in relation to context, recruitment and retention, reach, implementation and fidelity.

## Methods

The study was granted ethical approval from the Scotland A Research Ethics Committee (Reference 13/SS/229) and was registered as a trial with ISRCTN (ref: 50494254). In keeping with the Adults with Incapacity (Scotland) Act 2000, a participant with capacity provided their own informed consent, otherwise consent was provided by the nearest relative or welfare guardian.

The study design and methods have been published in detail as a protocol paper [22]. A brief outline of the Walk Well intervention is provided here followed by methods used for the process evaluation.

### The Walk Well intervention

Walk Well was a single-blind cluster RCT exploring the efficacy of a 12-week community based walking intervention for adults with intellectual disabilities (*n* = 102). The intervention consisted of three physical activity consultations (PAC) over a 12-week period with a walking advisor. The PAC method was refined and simplified to focus on four core behaviour change techniques: goal setting; self-monitoring; developing self-efficacy; and mobilising social support [22]. In addition to these four core elements the walking advisor tailored additional behaviour change techniques to the individual needs of each participant. In line with current physical activity recommendations [27] the walking advisor supported participants to develop a walking programme which aimed to increase walking by 30-minutes on at least five days per week, by week 12. Walk Well resources, including education booklets, a pedometer and step diary, were provided. The waiting list control group were advised to continue with their daily activity for 12-weeks, following which they were invited to participate in the Walk Well intervention. Data were collected at baseline, 12 and 24-weeks to assess change in: average steps walked per day; time spent in sedentary, light activity or moderate-vigorous intensity activity; Body Mass Index (BMI); waist circumference; and measures of subjective wellbeing [28–30].

### Context

The Walk Well study was designed, delivered and managed by a multidisciplinary team experienced in working with adults with intellectual disabilities. Team members included a manager within the local intellectual disability service and researchers experienced in walking interventions and behaviour change techniques. The manager of the local service provision played a key role in the study team, harnessing support from local day centres and provider organisations.

#### The process evaluation

A process evaluation was conducted based on guidance from Steckler and Linnan [26], the Medical Research Council [24], the RE-AIM framework [31], and the World Health Organisation [25]. The evaluation was performed by two researchers not directly involved in delivery of the intervention.

Five key elements were explored by the process evaluation, including: the intervention context; recruitment and retention; reach; implementation; and fidelity (defined in Table 1).

**Table 1:** Definition of the process evaluation outcome measures [24-26, 31]

Context	How external factors influence the delivery and functioning of an intervention.
Recruitment and retention	The factors associated with uptake and ongoing engagement with the intervention.
Reach	The extent to which the target audience comes into contact with the intervention.
Implementation	The structures, resources and processes through which delivery is achieved, and the quantity and quality of what is delivered.
Fidelity	The extent to which the intervention is delivered as conceived.

These factors were addressed using three methods on an ongoing basis during delivery of the intervention: (i) interviews with a variety of stakeholders; (ii) interviews with participants; and (iii) collation of miscellaneous information on a data input spreadsheet.

(i) *Interviews with stakeholders:* Firstly, in-depth semi-structured interviews were undertaken with a range of stakeholders (*n* = 6). The aim of these interviews was to gain insight from a variety of individuals involved in the study and included: the health professional delivering the intervention; the researcher responsible for delivery and day-to-day management of the intervention; two participants (one with positive study outcomes, one with no significant outcomes); the carer of a participant; and a manager from one of the local authority day centres. These 1-hour face-to-face interviews explored issues related to context, fidelity, and implementation, including the experiences of participating and/or supporting participation in the walking intervention.
(ii) * Interviews with participants:* Secondly, relevant process data were extracted from semi-structured interviews and focus groups conducted with 20 participants as part of a separate qualitative aspect of the study [32]. Interviews were undertaken with participants who did and did not have successful outcomes. Participants were asked to share their attitudes towards physical activity and walking, perceived benefits, drawbacks and impact of increased activity, subjective feelings of wellbeing, and any changes in view over the 12-week intervention. Overall, this qualitative data provided additional insight into the context and implementation of the intervention.
(iii) * Data input spreadsheet:* Finally, additional process data were extracted from the Walk Well data input spreadsheet which recorded multiple elements including: attendance, reasons for withdrawal from the study etc. This provided insight regarding recruitment, retention and reach of the intervention.

### Analysis

Interviews and focus groups were recorded and transcribed verbatim. Transcripts were analysed using criteria from a combination of process evaluation guidelines [24–26, 31]. This involved extracting key points related to the delivery of the intervention in practice. This data was interpreted in combination with information from the data input spreadsheet to present meaningful and useful information in relation to context, recruitment and retention, reach, implementation and fidelity.

## Results

### Main study outcomes

The main study outcomes have been published in detail elsewhere [23]. In brief, a total of 102 adults with intellectual disability participated in the Walk Well study (intervention group *n* = 54, control group *n* = 48). Baseline characteristics are presented in Table 2. At 12-week follow-up there were no significant within group or between group post-intervention effects on primary or secondary outcomes (Table 3).

**Table 2:** Baseline characteristics of intervention participants. Values are numbers (percentages) unless otherwise stated

Variable	Walk Well (54)
	N (%)
Female gender	25 (46 %)
Mean age (SD)	45 (14) years
Intellectual disabilities	
Mild	37 (70 %)
Moderate	11 (21 %)
Severe	5 (9 %)
Type of support	
Lives independently	3 (6 %)
Family carer	29 (53 %)
Paid carer	22 (41 %)
SIMD quintile	
0-20 % most deprived	36 (67 %)
20-40 %	8 (15 %)
40-60 %	5 (9 %)
60-80 %	2 (4 %)
80-100 % least deprived	3 (5 %)
Diagnosis of epilepsy	3 (6 %)
Visual impairment	24 (44 %)
Hearing impairment	11 (20 %)
Mental ill-health	15 (28 %)
Problem behaviours	9 (20 %)
Weight status (BMI kg/m^2^)	
Normal weight (18.5-24.9)	7 (14 %)
Overweight (25-29.9)	16 (31 %)
Obesity (30-39.9)	22 (42 %)
Morbid obesity (>40.0)	7 (14 %)
	Mean (SD)
Mean % time per day spent sedentary (SD)	64 (11)
Mean daily step count (SD)	4744 (2076)

*SD* standard deviation, *BMI* body mass index, *SIMD* Scottish index of multiple deprivation

**Table 3:** Main intention to treat analyses of effect of Walk Well programme on primary and secondary outcomes assessed immediately after end of programme (12 weeks)

	Walk Well		Control		Main between group comparison		
Outcomes	N	Mean (SD)	N	Mean (SD)	Mean difference at 12-weeks (95 % CI)^a^	p	ICC
Primary outcome							
Step count per day	42	4823 (2059)	40	4784 (2613)	69.5 (-1054 , 1193.3)	0.90	0.51
Secondary outcomes							
Percentage time per day MVPA	42	3.0 (2.6)	40	3.1 (2.1)	0.3 (-0.7 , 1.3)	0.55	0.42
Percentage time per day sedentary	42	66.4 (10.0)	40	65.9 (12.0)	1.6 (-3.0 , 6.1)	0.49	0.22
Total MET minutes per week	37	1311.9 (1293.2)	37	1154.8 (1103.7)	56.0 (-428.8,540.9)	0.82	0.02
Body Mass Index (kg/m^2^)	43	32.1 (7.7)	43	32.9 (7.5)	−0.2 (-0.83,0.41)	0.49	0.00
Waist circumference (cm)	45	104.9 (16.9)	42	107.8 (17.8)	−1.6 (-3.93,0.64)	0.15	0.00
Subjective vitality (mean score)	39	14.6 (2.5)	35	14.3 (2.8)	0.3 (-0.85, 1.52)	0.57	0.00
Self-efficacy (mean score)	43	14.4 (3.0)	42	13.7 (3.7)	0.8 (-0.68, 2.22)	0.29	0.08
EQ-5D (mean health utility score)	44	0.8 (0.27)	43	0.7 (0.30)	0.0 (-0.09, 0.14)	0.70	0.00

*SD* standard deviation, *ICC* intraclass correlation coefficient, *EQ-5D* European quality of Life 5 Dimensions, *MET* metabolic equivalents, *MVPA* moderate vigorous physical activity; ^a^Between group mixed effects model adjusted for cluster and baseline value

### Process evaluation outcomes

A range of useful and informative data was gathered from the process evaluation and is presented below in relation to context, recruitment and retention, reach, implementation and fidelity.

#### Context

Walk Well was delivered during a time of significant change within the local intellectual disability service. Support provision was affected by the closure of numerous day centres and changes to participant care packages. This resulted in low morale due to increased work pressure for day centre staff, increased demands on family carers, and disruption to established daily routines for adults with intellectual disabilities. Levels of study engagement by day centre staff and paid or family carers may have been negatively impacted by these changes. For example, staff working in day centres struggled to support study participants within time constraints.

*‘Because this is such a busy service and everything like that you can’t sit down [with a participant]. You know I would love to sit down with people and do that [support them].’ (Day centre staff)*

Workload pressures on staff also led to the end of existing walking groups within day centres.

*‘…we had a lot of individuals who were doing the project [Walk Well study] but we just didn’t have the staff to continue it [walking group] unfortunately.’ (Day centre staff)*

#### Recruitment and retention

Despite the complex changes to service provision the study employed a successful recruitment and retention strategy. 102 participants were recruited to the study, with attrition of only 19.6 % (*n* = 20). The number of participants lost to follow-up was similar in both the intervention and control group (22.2 % and 18.8 % respectively), with no significant differences in the characteristics of participants who completed or withdrew from the study.

Successful recruitment was attributable to several factors. Firstly, a multipoint recruitment strategy was employed, recruiting from three sources: day centres, provider organisations and health teams. The study team established links with key contacts and arranged to present study information, for example, at day centres and monthly health team meetings. A representative from the local authority disability service was part of the research team, and this was beneficial in gaining support from day centres and provider organisations. As such, a wide range of service staff were aware of the study and open to liaising for participant recruitment. Secondly, a personalised approach to study promotion was identified as a key factor. The walking advisor and study researcher interacted with potential participants in a one-to-one approach (carers/guardians were present for individuals with more severe intellectual disability), providing them with study information and a pedometer. An important element of Walk Well was to ensure adults with intellectual disabilities were treated equally and fairly. This involved giving them the opportunity to make an informed decision regarding their participation. The walking advisor and study researcher were experienced in working with this population and were sensitive to the issue of potential or perceived coercion.

*‘We used to go to day centres, talk to people on a one-to-one basis. It worked really really well and we could do a few talks [presentations] but the thing that worked the best was going to speak to people on their own on a one-to-one.’ (Walking advisor)*

*‘It kind of brought it [the project] to life and they could see what we would be doing with them and we showed them the pedometers.’ (Study researcher)*

Finally, participant retention was attributed to the friendly and approachable nature of the walking advisor and study researcher.

*‘I thought the two girls [sic][walking advisor and study researcher] were absolutely brilliant. I don’t think they could have done any better to be honest with you.’ (Paid carer 1)*

Retention was demonstrated by the collection of primary outcome data for 80.4 % (*n* = 82) at 12-week follow-up. The communication skills and friendly nature of the walking advisor and study researcher were again identified as contributing factors. The ability to set goals and self-monitor (via use of the pedometers and step diaries) was also highlighted as a factor for retention. Carers commented on the enjoyment that participants received from wearing their pedometer, recording their step counts and monitoring their progress. Participants were also motivated to work towards their end of study certificate.

*‘We could see [the step count] and say “Look how many steps you did today”. If he got higher the next day he was quite proud of himself.’ (Paid carer 1)*

*‘He enjoyed the fact that he reached his goal to get the certificate. That’s right, he completed something. He always likes to finish things when he starts them. Sometimes in X’s life it is difficult for him to complete things.’ (Family carer)*

The important issue of ‘freedom of choice’ was raised in relation to those participants who withdrew from the study. Although the majority of participants were perceived as eager to join, one participant commented on ‘pestering’ or ‘nagging’ by day centre staff to take part. This raises an important issue in relation to not only retention but to participant choice.

*‘For some people they were really encouraged by carers or the day centre staff and I wouldn’t say forced, but they were almost strongly encouraged to do it. I think for people who were kind of encouraged to do it, as soon as they were given the opportunity to not do it anymore they just dropped out. I don’t think there was really anyone that has finished the intervention that really didn’t want to do it.’ (Study researcher)*

*‘They were kind of pestering me in a way … it was like being nagged at so I said “Yes, I will do it” and try something new.’ (Participant 1)*

#### Reach

The successful recruitment strategy reached a sample of participants who were representative of adults with intellectual disabilities: 91 % (*n* = 93) had mild or moderate intellectual disability; 67 % (*n* = 71) lived in the most deprived quintile as measured by the Scottish Index of Multiple Deprivation [33]; 58 % (*n* = 59) had a BMI ≥ 30; the majority of participants were sedentary with a mean daily step count of < 5000 steps; and the majority had additional health needs e.g. 55 % (*n* = 55) with visual impairment. There was an even distribution of participants with family carer support compared with paid carer support (51 % (*n* = 52) and 44 % (*n* = 45) respectively) (Table 2).

#### Implementation

Numerous factors were identified which positively and negatively affected implementation of the Walk Well intervention.

Firstly, level of carer engagement was identified as a key facilitator of walking participation. Pro-active and consistent carer engagement was perceived by the walking advisor to be associated with successful participation in Walk Well. In these instances, participants were supported in several domains, including: the provision of ‘options’ for walking routes; the integration of walking as part of participants’ day to day activities; providing incentives for walking participation; and the provision of ongoing encouragement, positive feedback and support with self-monitoring.

*‘I usually take my phone and put the music on and he kind of marches to the music which is quite good.’ (Paid carer 1)*

*‘[We supported him] by encouraging him and asking him how he got on and did he enjoy it and he said really good, he was enjoying and we got feedback from where he went and all sort of stuff.’ (Family carer)*

*‘Sometimes X used to help me and some of the staff [helped me] because I might trip up and some of them were walking slow and I am not a slow walker.’ (Participant 2)*

The walking advisor observed higher levels of engagement when the carer was a family member. Paid carer engagement was perhaps affected by low morale due to closure of day centres and changes in service provision. Successful paid carer engagement was, however, demonstrated in several circumstances where daily walking had been incorporated into a participant’s official support plan. Carers reported how physical activity was discussed at support team meetings, and written into individual support plans. In all circumstances the decision was made in conjunction with input and agreement from the individual with intellectual disabilities.

*‘That time is set aside for her. That walk is her time, it is nothing to do with anyone else. The only way she wouldn’t make the [walking] group is if it was a holiday or she was unwell.’ (Paid carer 2)*

In contrast, participants who were inconsistently supported by their carers were perceived to have less successful participation in Walk Well. Several explanations were presented for poor engagement by carers, including: low morale and changes in the working environment; the burden of paperwork and other administrative duties for participants; lack of consistency in shift patterns; lack of communication between carers; and being responsible for more than one individual with intellectual disabilities.

*‘It depends on the carer and their level of commitment. I think we came into it at a bad time with privatisation and morale was very very bad in quite a few day centres that we went to.’ (Walking advisor)*

*‘I was coming in sometimes [to support participant] and they had new staff in and they didn’t know how to do it [support participant and complete step diary]. So I was coming in and doing the seven day [diary] … and I didn’t know if he had done his walk that day or the previous few days so I couldn’t tick it or whatever.’ (Paid carer 2)*

*‘[Paid carers] are so understaffed they work all the hours and the last thing they need is someone like me going “Ah let’s see some walking.” (Walking advisor)*

Secondly, overall the use of pedometers and Walk Well resources was viewed positively. Pedometers were used for self-monitoring, goal setting and as a motivational tool. Although some participants disliked it, the majority of participants seemed to enjoy wearing the pedometer and were aware that it recorded their daily activity.

*‘She absolutely loved her pedometer, so she would wear it around her neck. She didn’t want to put it in her pocket, she wanted everyone to see it. So her sister put in a chain for her so she could have it on her neck and show everyone. So people would ask “What’s that?” and she would say “That’s for my walking.” (Study researcher)*

*‘Sometimes I get up in the morning and I walk from the bedroom to here and I have done 200 steps.’ (Participant 3)*

*He gives it [pedometer and step diary] to you at night time when he is getting into his jammies. He gives you it to mark down in the book and he will remind you to mark it down.’ (Paid carer 2)*

Although pedometer wear was positive and motivational for some, not all participants *understood* what the step count meant and how this could be used to progress their walking. For these participants the step diary could be perceived as intimidating. An alternative ‘tick box’ resource was developed by the study team so that individuals experiencing difficulty with the step diary could still engage in self-monitoring behaviour.

*‘Even if they didn’t understand the numbers on the screen, they maybe didn’t know if 10,000 steps was good or bad, and some people maybe didn’t know what the number meant. But I think everybody, no matter what their level of disability, had a sense of awareness that the pedometer was related to their walking.’ (Study researcher)*

*‘I would ask them to do ticks [on the days they had walked] and put it on the wall or fridge so that everybody can mark it, even put a smiley face. It is a good motivation tool. Well I think it worked for people who have found the walking diary quite intimidating if you like.’ (Walking advisor)*

Other resources, such as the participant booklets, were used by the walking advisor during the physical activity consultations to provide and reinforce information. Resources, such as the booklet, were designed to engage participants with relevant use of visual images and appropriate text.

*‘The pedometer walk [demonstration walk during initial consultation] was very good. I think that really worked as people wanted to see how it works etc… just going through the book as well, it was a good book …and then afterwards I ask them a question so we can reiterate it that way as well.’ (Walking advisor)*

Thirdly, despite not being mentioned at the recruitment stage many day centres, participants and carers assumed the Walk Well study involved the provision of a walking group. This perhaps led to disengagement and reduced enthusiasm upon the absence of a walking group.

*‘I do think people expected us to be a walking group and I think that was a bit of a disappointment to them, and although I gave them some health walks brochures and I contacted health walks for them it was not the same.’ (Walking advisor)*

Local walking groups (via the organisation *Health Walks)* were, however, promoted to all participants. The enjoyment and benefits of walking groups were highlighted by both carers and participants.

*‘The best thing about supporting X is when she said “I enjoyed that” at the end or “I have had a good day” and sometimes she does say that in the walking group.’ (Paid carer 2)*

*‘As I kind of enjoy it and when meeting others when everyone is out [in the walking group], I can walk more than when I walked then.’ (Participant 4)*

#### Fidelity

The physical activity consultations were refined and streamlined for use with adults with intellectual disability. This involved focusing on several core components and having the flexible option of incorporating additional behaviour change techniques as and when needed. The walking advisor reported that overall the physical activity consultations worked well. She was able to engage participants in some, if not all, elements of the consultation. A key factor of delivering the physical activity consultations was flexibility, with all consultations tailored to the individual needs of the participant. Carers had the option to contribute where appropriate.

Several challenges were highlighted by the walking advisor including: finding effective ways to involve participants who had communication impairments; the volume of questions; and the length of time in between follow-up consultations.

*[The success of the consultations] it will depend on the communication skill s of the participants. I think they were well received. There are too many questions but you can reduce those questions by amalgamating questions and then get back to our leaflets to look at visual things.’ (Walking advisor)*

*‘The main [challenge] is communication, again sometimes people weren’t understanding what I was saying. Sometimes I think it may have worked better if it had been with [people with] not quite so severe learning [intellectual] disabilities, because that is difficult to work with and it is soley the carer that is doing that.’ (Walking advisor)*

*‘…I think six weeks in between [consultations]. I had given them the choice to ring me but I have not to ring them. That is a long time.’ (Walking advisor)*

Feedback from participants, carers and the study team identified the participant burden of the data collection questionnaires. One adaptation was therefore made to the protocol to reduce the number of questionnaires used during data collection. This involved removal of two questionnaires (the Index of Community Involvement and Index of Participation in Domestic Life) which reduced data collection by approximately 15-20 minutes.

*‘He said he would try it [Walk Well study] but when the researcher came to the house it was a lot of questions [baseline data collection]. So he got quite “No I can’t be bothered with this anymore”.’ (Paid carer 1)*

*‘We decided to cut down some of the questionnaires because they weren’t really key outcomes of the study. I would be there for over an hour and it was taking up quite a big part of their day. When we took those measures out it significantly reduced the data collection.’ (Study researcher)*

In general, the use of accelerometers as a data collection tool was feasible and well accepted by participants, demonstrated by 80.4 % (*n* = 82) of participants providing accelerometer data at 12-week follow-up. However, some participants found it uncomfortable and there were issues of obtaining sufficient data over a 7-day period. It was suggested by the study team that in future studies participants could be asked to wear the accelerometer for two weeks in order to gather sufficient data in one period.

## Discussion

This is the first process evaluation of a community-based walking intervention for adults with intellectual disabilities. Although Walk Well was not effective in increasing physical activity or improving health outcomes, our process evaluation has identified several important considerations for future interventions.

**Firstly, a multipoint recruitment strategy** employing personal one-to-one interaction with potential participants was a feasible and effective method of recruitment for adults with intellectual disabilities. Accessing participants via day centres and service providers recruited a sample representative of the wider adult intellectual disability population [34]. In addition to gender, age and health status the Walk Well sample also reached vulnerable adults at high risk of health problems i.e. high BMI, physically inactive and sedentary lifestyle. Retention of 80.4 % was similar to that of other 12-week walking studies in the general population [35] and lifestyle interventions for adults with intellectual disabilities [36]. Retention may also have been supported by reducing the participant burden of data collection questionnaires. A previous systematic review of recruitment strategies for walking interventions found that publications lacked information on the effectiveness of their recruitment methods making it difficult for researchers to use appropriate methods in future studies [37]. Our findings address this gap by demonstrating that a multipoint recruitment strategy for community based walking interventions can reach individuals who may benefit from physical activity behaviour change.

**Secondly, consistent input and engagement from carers** was a key factor in the success of participants’ behaviour change. Walk Well was designed to harness autonomy and motivation of the individuals with intellectual disabilities. However, to effectively engage in walking behaviour the majority of participants required support from family or paid carers. Melville et al [38] suggests that carers may have limited knowledge of healthy lifestyles and therefore require information and support to effectively support participants. Although social support was a key component of the programme model, and a specific written resource was developed for carers, perhaps a more formal strategy, or specific programme components, were needed to inform day centres, paid carers or family carers on how to effectively support participants in their walking programme. Carers who were not involved in the PAC also received limited feedback from participant’s consultations with the walking advisor. This led to only the most engaged carers exploring participant goals, action plans and resources. Low morale and significant changes in service provision also appeared to negatively impact carer engagement, highlighting the importance of the social and environmental context of community based interventions. In contrast to Walk Well, which focused on engaging participants in their own behaviour change, a study undertaken in Sweden focused on changing the behaviour of paid carers in group care homes [39]. Their findings demonstrated a significant intervention effect on the physical activity levels of adults with intellectual disabilities (increase of 1203 steps/ day, *p* = 0.039). Although the characteristics of the sample and intervention are not directly comparable to Walk Well they do highlight the need for effective methods of engaging carers in the behaviour change process. This is an important consideration for future research.

**Thirdly, physical activity consultation** had variable success engaging adults with intellectual disabilities in discussion about behaviour change. Consultations worked well for the majority of participants who demonstrated good communication skills (*n* = 48), but were challenging for the few individuals with more severe intellectual disabilities (*n* = 5). Overall, this was an encouraging finding to be explored with further research. Despite the consultations being generally well received the intervention was not effective. Although there were no significant changes in daily step count or physical activity levels it was challenging to interpret the frequency, duration and intensity of *individual* walking bouts. This would be interesting to explore to help assess whether there were any such changes. It is possible that the level of cognition required for effective behaviour change requires an intervention of longer than 12-weeks duration. Twelve week interventions have been effective in improving physical activity levels within the general population [35, 40], however, adults with intellectual disabilities do experience greater and more complex barriers [41, 42]. Although Walk Well is ineffective over 12-weeks it is worth exploring longer durations to give time for the walking consultations to address the greater barriers faced by this population. A greater frequency of contact between the walking advisor and participant could also be explored. This was observed by the walking advisor who suggested that participants could benefit from an additional consultation between baseline and 6-weeks to reinforce goal setting and action plans.

**Fourthly, pedometers complemented the core components** of the physical activity consultation and were a feasible method of motivation, goal setting and self-monitoring. However, not all adults with intellectual disabilities were able to interpret the step count and understand its purpose, reinforcing our finding that adults with intellectual disabilities often require ongoing, and consistent, support to effectively set goals and self-monitor their walking behaviour. The important role of support was also highlighted by disappointment in the lack of provision of a walking group. This disappointment, by both participants and carers, perhaps identifies a need in this population for social support and interaction with peers. Social support and self-efficacy are known to predict physical activity behaviour in individuals with intellectual disability; and in those of adult age support comes from both staff and peers [42]. Social support can also lead to sustainable behaviour change [43]. Walking groups are therefore a potential method of promoting walking in this group.

**Finally, the complex issue of ‘freedom of choice’** needs careful consideration for adults with intellectual disabilities. One participant reported feeling ‘pestered’ or ‘nagged’ by their day centre staff into joining the study. In line with Good Clinical Practice [44] no individual should feel pressured to participate in research. Autonomy is one of the four cornerstones of good ethical conduct; along with beneficence, non-maleficence and justice [45]. However, exercising autonomy and reaching an informed decision requires deliberation, understanding and decisional balance; cognitive skills that some adults with intellectual disabilities may not have [45]. To make an informed lifestyle choice they need to effectively consider the benefits and risks of positive and negative behaviour [46]. This led to debate by carers on ‘freedom of choice’ and ‘health improvement’ i.e. Was encouraging individuals who lacked motivation a bad thing? Or was not encouraging individuals, because they lacked motivation, a bad thing? This uncertainty over ‘freedom of choice’ was demonstrated in our study where participants, supported by carers, were sometimes entrenched in poor lifestyle behaviours, for example, eating several boxes of chocolates or using a taxi for a distance of 200m. Previous research in Glasgow has shown that service providers typically organise taxis for people with intellectual disabilities travelling even very short distances [47]. Findings from previous research and discussion within our study team and with Walk Well carers reflected the complexity of this issue. Opinions were varied including: (i) that participant choice was essential even when it led to long-term unhealthy lifestyle behaviours [48]; (ii) there was disagreement between individuals regarding use of the term ‘encouraged’ versus ‘pressured to participate’; and (iii) that not encouraging individuals to participate in healthy behaviour, because of their lack of interest or motivation, may in itself be harmful and lacking of care [49]. Future studies should ensure, as this one certainly aimed to do, that adults with intellectual disabilities are provided with information in a way that allows them to deliberate the benefits and risks of participation (not just in research, but also in new lifestyle behaviours), with emphasis that participation is entirely voluntary and they are free to withdraw from the study at any time without negative impact on their care or personal relationships.

## Conclusions

Walk Well was based on previous successful interventions in the general population but was not effective in its current form for adults with intellectual disabilities. It was, however, shown to be a feasible and acceptable method of engaging adults with intellectual disabilities in activities to support physical activity behaviour change. Significant health inequalities exist between this group and the general population so finding methods of effectively adapting physical activity interventions for this group is important. Participants may require an intervention of longer than 12-weeks, with greater frequency of contact to support effective behaviour change. Future studies need to implement strategies to maximise social support by engaging carers in the behaviour change process and providing opportunities for interaction with peers.

### Strengths and limitations

This is the first process evaluation exploring the implementation of a walking intervention for adults with intellectual disabilities. Findings represent the unique contextual and environmental factors of service provision in the city of Glasgow, Scotland. As such, they may not be representative of service provision in other areas.

## Abbreviations

BMI, body mass index; PAC, physical activity consultation; RCT, randomised controlled trial
